# Effect of Biochar and Inorganic Fertilizer on the Soil Properties and Growth and Yield of Onion (*Allium cepa*) in Tropical Ethiopia

**DOI:** 10.1155/2021/5582697

**Published:** 2021-08-30

**Authors:** Abreham Berta Aneseyee, Tekilil Wolde

**Affiliations:** Department of Natural Resource Management, College of Agriculture and Natural Resource, Wolkite University, Wolkite, Ethiopia

## Abstract

Biochar is a carbon-rich product, which is processed by pyrolyzing biomass to improve soil properties and maintain environmental sustainability. This study aim was to investigate the effect of biochar and inorganic fertilizer on soil properties, growth, and yield. Four treatments and four replications have been used for the experimental plots using Randomized Complete Block Design (RCBD). Soil physiochemical properties have been investigated based on soil samples within 0–30 cm depth in each plot. The two types of biochar (grass and chat waste) have been used for the treatments. The pyrolyzing temperature required for grass and chat waste is 250°C and 1100°C, respectively. The plant height, total yield, and the marketable and nonmarketable yield of onion have been examined. The analysis showed that treatment with grass biochar and inorganic fertilizer had a similar effect on soil properties but chat (C*atha edulis*) biochar had a lower effect on soil properties. The total yield and days to 70% maturity have been increased due to the cumulative treatment of grass biochar and inorganic fertilizer. The biochar of grass and inorganic fertilizer have been significantly increased in marketable bulb yield, but unmarketable yield becomes decreased. The chat waste and controlled treatments shown an increased unmarketable yield and declined marketable bulb yield. Overall, biochar can substitute the inorganic fertilizer, which can help to improve the *w* soil fertility and environmental sustainability. Therefore, biochar has a win-win solution.

## 1. Introduction

Biochar is helpful to improve soil nutrients and enhances soil quality [1, 2]. It is a carbonaceous residue generated by heating biomass in the absence of oxygen, with a process of pyrolysis, which transforms organic matter into a solid residue (biochar) and a vapour [3, 4].

Several studies have been conducted on biochar researches, focused on the effects of biochar in tropical soils, indicating improved plant growth, increased soil nitrogen retention, bioavailability, and plant uptake of supplemented nutrients [5, 6]. However, many of these researches have been undertaken in the laboratory without field testing of crop planting [7]. According to Wang et al. [8], biochar applied to soils has a significant environmental benefit such as soil carbon sequestration and agricultural benefits by increasing the soil fertility, which leads to maximizing the yield of the crops [9, 10].

The feedstock for biochar could be agriculture and forestry such as wood chip and wood pellets, tree bark, crop residues (including straw, nutshells and rice hulls), switchgrass, organic waste including distiller grain, bagasse from the sugarcane industry, and olive waste [11, 12]. Hence, biochar production can be useful to maintain waste disposal challenges [13] by keeping them out of landfills and burning, which enables converting them into usable resources. All feedstocks used for making biochar could not show a similar effect on soil properties and crop productivity. For example, the agricultural feedstock can improve soil fertility more, which might be enhanced plant growth and crop yield [7].

Biochar is a simple solution to alleviate the environmental problems and concurrently increase crop yield. Liu et al. [7] and Jeffery et al. [14] reported that biochar increased crop productivity by 11% in a variety of crops. According to Agegnehu et al. [15] and Subedi et al. [16], crop biomass and yield have been increased. The soil was the main factor for crop yield changes due to the application of biochar. For example, Jeffery et al. [17] investigated that biochar has been generated an increase of 25% in yield in acidic soil, and also there has been an increase of maize productivity by 7.5% due to biochar application [18]. Brown et al. [19] review report also shows an increase in maize yield by 19% across many studies due to the application of biochar.

The best altitude for growing onions under Ethiopian conditions is between 500 and 1500 m above sea level. Statistics on the production of onion in Ethiopia showed that about 15, 290 ha of the land was cultivated and 0.2 million tons of bulbs were produced in the year 2001/2002 [20]. However, average attainable yields have been declined due to soil fertility problems; continuous use of inorganic fertilizer and inappropriate soil fertility management practices are among the major factors limiting the productivity of onion in Ethiopia [21].

The study site has sufficient and high availability of the feedstocks for biochar making and grass has been excessively found in the study area but farmer's burn it during the dry season to rejuvenate new grass for their livestock feeding, which release a large amount of Green House Gases (GHGs) such as carbon dioxide (CO_2_)_,_ methane (CH_4_), and other trace gases to the atmosphere.

Most of the people in the study area have engaged in agriculture practices and they need to buy inorganic fertilizers for their crop farming, but this is not easily affordable by the entire farmers since inorganic fertilizer is expensive, overpriced for the local farmers and it is not viable for environmental conservation. Therefore, this research provides an option for the substitution of inorganic fertilizer by new technology, biochar.

Chat waste is also generated in the town which is thrown into the surrounding environment that pollutes the air, water, soil, and human health so that the feedstock of grass and chat waste using a pyrolyzing process help to keep the sustainability of the environment and improve agricultural productivity.

The objective of this study is (1) to evaluate the effect of biochar and inorganic fertilizer on soil properties and (2) to investigate the effect of grass and chat biochar on the growth and yield of onion.

## 2. Materials and Methods

**Figure 1:**
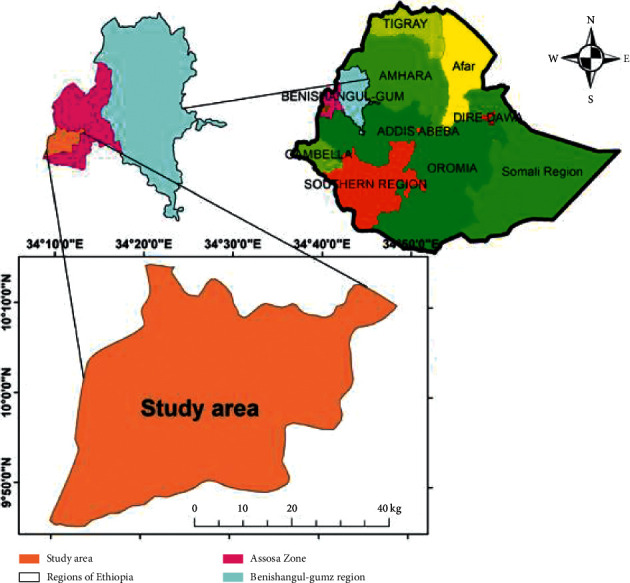
Map of the study area.

This research was conducted in western Ethiopia, Benishangul Gumuz region (BGNRS), in Assosa zone at Amba 2 Kebele, which is 5 km far from Assosa town (Figure 1). The study area latitude is 9°45′ 0′′ and longitude is 34°10′ 0′′, with an elevation of 1570 meters. The soil type of the study area was characterized as Nitisol [22].

### 2.1. Biochar Preparation

**Figure 2:**
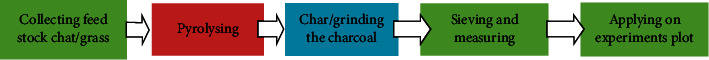
Biochar making process.

Two types of locally available biochar were used in this study. The first type was grass feedstock, which was collected from the grazing land in the Assosa zone of Ethiopia. The grass biochar was prepared at 250°C for 10 hours using furnaces through the process of pyrolysis based on the recommendation of Warnock et al. [23] and Sohi et al. [24] (Figure 2) and the second was chat waste, collected in Assosa town, pyrolysis at 1100°C for 4 hours. The details of biochar making procedures are also provided by Novak et al. [12] and Sigua et al. [25]. After the pyrolysis of both processes, the biochar was ground into small granules and passed through a 2 mm sieve to have the same particle size and easily mix in the soil.

### 2.2. Composition of Biochar

The biochar composition analysis was undertaken at JIJ Laboratory, Ethiopia (https://jijelaboglassplc.com/). The pH was measured using FAO potentiometer using a ratio of 1 : 2.5 soil-water ratio using a glass-calomel combination electrode [26]. Electric Conductivity (EC) was determined by FAO-conductivity-water extract and Cation Exchange Capacity (CEC), and exchangeable cations (Na and K) were assessed using FAO principles of sodium equivalent by flame photometer. The exchangeable cations (Ca and Mg) were determined using ammonium acetate extract-EDTA titration. Nitrogen and organic carbon were analyzed using the Black–Walkley method [27]. Sulfur was analyzed using the turbid metric method, and phosphorus was also analyzed according to the molybdenum blue colour method, with atomic absorption spectrophotometer calorimetric [28].

### 2.3. Experimental Design

**Figure 3:**
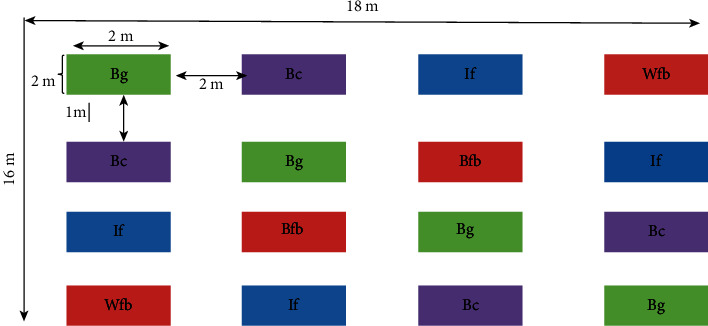
The layout of the experiment.

**Figure 4:**
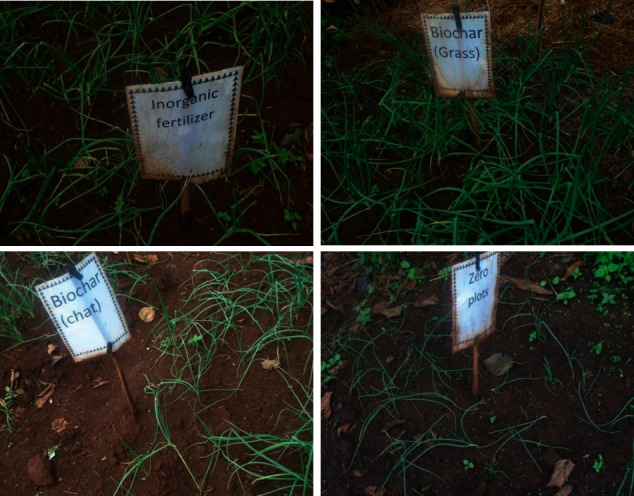
Field experiment and its treatments.

Each treatment has been applied on the plot size of 2 × 2 m (4 m^2^), separated by a distance of 2 m between a block and 1 m within a plot [29] (Figures 3 and 4). The experiment consists of four treatments with four replications arranged in Random Complete Block Design (RCBD). A total of 16 plots, with an area of 288 m^2^, were prepared in the field. A recommended 0.5 kg/m^2^ of biochar grass (Bg) and chat (Bc) was applied in the treatment plots [23] and treated plots with inorganic fertilizer (If) recommended 150 kg/ha urea and 100 kg/ha DAP was also applied to the soil [30] and there was a control plot (Wfb)—free from biochar and fertilizer.

### 2.4. Data Collection of Crop Growth and Yield

The crop growth and crop yield performance are depending on the plant maturity and the quality of the yield. Therefore, to determine the effect of biochar on the growth and yield of onion, days to 70% maturity, height, total yield, and marketable and unmarketable yield data have been collected from each plot.

- Plant height (cm): data of plant height at initial, mid, development, and late stages have been measured. Measurements had been undertaken from the ground level to the top of the plant after transplanting and at the maturity of the plant.
- Days to 70% maturity: it is the total number of days from emergence until 70% of the plants has attained physiological maturity. It is the time needed for the plant to reach maturity for harvesting. Plant maturity informs us about how long it takes from the time the seed is sown to the point when the plant is ready to set bulb. The ready bulb or the fruit cannot damage unless some emergency climatic risk such as high rainfall and large hail.
- Weight of marketable fresh bulb yield (t/kg): it is the weight of healthy and marketable bulbs that can be consumed by a human after harvesting and it has been measured using a sensitive balance after harvesting of onion. Total weight of clean, diseased, and damage-free bulbs with greater than 21 grams in weight was considered as marketable bulb yield. This has occurred due to the sufficient presence of fertilizer and nutrients.
- Unmarketable fresh bulb yield weight (t/kg): the weight of decayed, insect-attacked, and abnormal bulbs including multiple bulbs, thick-necked bulbs, and too small bulbs having less than 20-gram weight during harvesting were also measured using sensitive balance.
- Total yield (t/ha): it is the sum of the weight of marketable fresh bulb yield and unmarketable fresh bulb yield.

### 2.5. Soil Sampling and Analysis

Before planting, disturbed composite soil samples have been taken from the entire designed experimental plots, and this has been done after harvesting of onion to analyze the soil physiochemical properties by following appropriate laboratories procedures. That is, before and after the application of biochar and fertilizer, soil samples have been taken at a depth of 0–30 cm in each selected plot. The soil sample was packed using plastic in the field. After sampling, the following parameters were determined: pH (FAO potentiometer using a ratio of 1 : 2.5 soil-water ratio), electric conductivity (EC) (FAO-conductivity-water extract), cation exchange capacity (CEC), and exchangeable cations (Na and K) (FAO principles of sodium equivalent by flame photometer), nitrogen and organic carbon (Black–Walkley method), sulfur (turbid metric method), and phosphorus (molybdenum blue colour method).

### 2.6. Data Analysis

After the data collection was completed, the data was organized and recorded on the Excel datasheet for analysis. The recorded data were analyzed using R software, and a 5% significant level was tested using one-way ANOVA. Box plots are also used to show a graphic description of the data.

## 3. Results and Discussion

### 3.1. Composition and Characterization of the Biochar

The characteristics of the biochar derived from grass and chat waste are shown in Table 1. The composition of biochar has a significant difference due to the variation of the feedstock. For example, grass biochar showed a higher pH than chat waste biochar and CEC and EC are also higher in grass biochar, comparing to chat waste. The chemical analyses of the feedstock biomass of grass revealed that the highest amount of organic matter (6.7 ± 1.2), while chat biochar contained the lowest (1.5 ± 0.07). Similarly, sodium, potassium, magnesium, nitrogen, sulfur, and phosphorus contents of the grass biochar were higher as compared to chat biochar.

### 3.2. Soil Characteristics

The soil properties become increased due to the application of biochar to the soil [31]. An increase in soil parameters as a result of biochar and inorganic fertilizer amendment has been similarly reported by several researchers [32, 33]. Biochar and inorganic fertilizer did not show a significant difference in crop yield and agronomic structures [31]. This is a similar finding to our study; that is, onion yield is similar when treated by biochar grass and inorganic fertilizer (Tables 2–6).

#### 3.2.1. pH

Soil pH became less acidic when biochar has applied [34]. In this study, the pH was significantly different among the treatments and its range varied from 4.90 ± 0.8 to 6.74 ± 1.0. The highest pH was 6.74 ± 1.0, which was found in the experimental plot having inorganic fertilizer treatment and grass biochar treatment. The lowest pH (4.90 ± 0.8) was found in the controlled treatment (Table 2). Statistically, the pH of biochar grass and inorganic fertilizer treatments has no significant differences; similarly, chat waste and control treatments also have no significant difference in pH due to the properties of the chat waste. However, chat waste treatments show a statistically significant difference with the treatment of grass biochar and inorganic fertilizer (*p* < 0.05). Therefore, adding grass biochar to the soil can decline acidic soil.

#### 3.2.2. Electric Conductivity (EC) and Cation Exchange Capacity (CEC)

The study shows that biochar increased CEC, due to the existence of cation exchange sites on the surface of biochar [14, 23, 35]. The EC was lowest (0.2 ± 0.02 dSm^−1^) in the control treatment and the highest was 0.33 ± 0.00 dSm^−1^ in grass biochar. The CEC shows a similar pattern with EC in all treatments plot (Table 2).

#### 3.2.3. Soil Organic Matter (SOM)

Applying biochar to the soil, increased soil C thus contributes to the sequestration of carbon. Much of this carbon sequestration is due to the inert portions of the biochar material [34]. The soil organic matter was higher in biochar treatment as compared to inorganic fertilizer, chat, and controlled treatment, implying that the soil carbon sequestration of biochar grass is higher than inorganic fertilizer, chat, and controlled treatment. Therefore, using biochar contribute to smart agriculture occurrence as well as mitigation of climate change by sequestering carbon in the soil.

Soil organic matter (SOM) has been varied across the treatments (Table 3). For example, SOM in biochar grass has a significant difference with the controlled treatment. The effect of inorganic fertilizer and biochar of grass on the addition of SOM was consistent, but the biochar of grass exerted considerable influence overall treatment on the accumulation of SOM. The values of SOM increased with adding biochar as compared to inorganic fertilizer since there was a significant difference between the two treatments (*p* < 0.05). Since SOM is directly proportional to the percent of organic carbon, this indicates that the soil carbon sequestration is higher in grass biochar. This shows that the biochar of grass can contribute to climate change mitigation in addition to improving soil fertility.

#### 3.2.4. Soil Nitrogen (N)

Soil N did not show a significant difference in the treatment of inorganic fertilizer and biochar grass (*p* > 0.05), but it had a significant difference between biochar grass with the control and chat plot (*p* < 0.05). In other words, the soil N is greater in a plot of biochar grass and inorganic fertilizer treatment but lower in the plot of biochar of chat and controlled treatment. The results suggested that both biochar grass and inorganic fertilizer had a considerable influence on the accumulation of soil N.

#### 3.2.5. Metallic Ions (Exchangeable Cation)

The results show that biochar of grass and inorganic fertilizer treatment has been an equal effect on the chemical properties of soil properties. Biochar increased the K and Na availability as it is a source of the cations [36]. Sodium (Na) has affected equally with the treatment of biochar of grass and inorganic fertilizer (*p* > 0.05) while it was varied with the treatment of chat and controlled treatment (*p* < 0.05). This was also similar for potassium and magnesium (Table 4).

Therefore, the study analysis shows that the biochar of grass enhances pH, nitrogen, phosphorous, CEC, EC, SOM, and exchangeable cations (Ca, Mg, and Na). This is supported by Kamara et al. [37], which shows an increase in pH, reduced bulk density, increased cation exchange capacity, and nutrient availability in the rice straw biochar application. Therefore, the analysis of the study shows that biochar grass and inorganic fertilizer could enhance the yield of onion and soil quality.

### 3.3. Agronomic Characteristics

The biochar treatments were found to increase yield, the final biomass, root biomass, and plant height in comparison with controlled treatments [38]. Thus, the research finding indicated that the yield of onion and its height treated by biochar was higher than the control treatment but equal in effect with inorganic fertilizer. The plant height at a late stage using grass biochar and chat biochar treatment has been recorded as 58.6 ± 0.08 cm and 49.5 ± 0.14 cm, respectively. This is a significant difference in the height of the crop due to the application of different treatments, but grass biochar and inorganic fertilizer plant height have a similar height (Table 5).

#### 3.3.1. Days to 70% Maturity

There was a significant effect (*p* ≤ 0.05) on days to 70% maturity of the crop due to the application of biochar grass and inorganic fertilizer compared to biochar of chat and control plot (Table 5). The highest number of days to 70% maturity was recorded in plots with biochar of grass (117.3 ± 0.13 days) and the lowest (102.5 ± 0.33 days) was recorded in plots of biochar chat treatment. The result indicated that days to maturity were prolonged in response to increased levels of treatment application. This may be attributed to the role those different treatments play in promoting vegetative growth before the start of bulb development. However, the application of biochar of chat had not a significant difference (*p* > 0.05) on days of plant maturity compared to controlled treatment plots.

#### 3.3.2. Plant Height

The highest plant height was recorded in inorganic fertilizer treatment (60.0 ± 0.21 cm) followed by biochar grass treatment (58 cm) while the lowest was recorded in controlled treatment (20 cm) (Table 5).

A significant variation of plant height (*p* ≤ 0.05) in plots with biochar of chat and inorganic fertilizer or biochar of grass was observed. Biochar grass treatment shows a higher plant height as compared to controlled treatment. Plant height was also significantly different in each growth stage, but no significant height difference was found during development and late stages in the treatment of biochar grass and inorganic fertilizer. This could be due to the crop being underway for the development of the bulb and growth of the plant was ceased during this stage.

#### 3.3.3. Marketable Fresh Bulb Yield (MY)

The analysis indicated that the effects of biochar and inorganic fertilizer show insignificant change on marketable fresh bulb yield (*p* < 0.05) (Table 5). The biochar of grass and inorganic fertilizer were required to realize significant and similar increases in fresh marketable bulb yield of onion on the experimental soil. Therefore, the highest (28.96 t/ha) marketable yield was recorded on a plot with inorganic fertilizer whereas the lowest (25.30 t/ha) was observed on a plot with biochar of chat treatment.

#### 3.3.4. Unmarketable Yield (UNMY)

Small-sized bulbs were produced in the untreated plots due to nutrient deficiency. The highest (8.14 ± 0.02 t/ha) unmarketable bulb yield was recorded on a plot of chat treatment followed by controlled treatment, but the lowest (2.82 t/ha) was recorded in a plot with the treatment of inorganic fertilizer followed by biochar of grass (2.93 ± 0.02) (Table 6).

#### 3.3.5. Total Yield (TY)

Many research reports show that the treatment contains biochar as a soil amendment is higher than any other treatment. For instance, wheat yield treated with biochar was significantly increased by 15.7% and 16.5%, respectively, over the inorganic fertilizer applications [39]. However, the treatment with biochar of grass and inorganic fertilizer had an equal effect on the yield of onion. This shows that biochar has a huge capacity to retain nutrients and holding water in the soil to improve crop productivity.

Total yield has also varied among the treatments (Table 6). The highest TY was recorded in inorganic fertilizer (31.78 ± 0.02 t/ha) followed by biochar of grass (31.17 ± 0.06 t/ha). However, TY obtained from inorganic fertilizer and biochar grass is nearly similar, with a difference of 0.61 ± 0.04 t/ha. According to [34], applying biochar reduced the leaching of soil and maximized crop yield. The lowest TY was 24.44 ± 0.02 t/ha in the treatment of biochar chat, followed by controlled treatment. The research finding shows that the tomato yield increased after biochar applications [40].

Many research findings show that biochar amendment was better than fertilizer but it has evidence that a synergistic effect of biochar and fertilizer on the soil [34]. The study indicated that biochar grass has no different effect on inorganic fertilizer on the yield of onion and soil properties. These indicated that biochar can substitute inorganic fertilizer, which is helpful for the local farmer by providing nutrient-rich fertilizer (biochar) that produces local affordable feedstock and waste materials.

## 4. Conclusion

Pyrolysis biochar production has been undertaken based on the feedstocks of grass and chat waste collected locally in the study area. The study indicated that the application of grass biochar and inorganic fertilizer has improved soil properties and yield of onion as compared to chat biochar and controlled treatment. The analysis also indicated that the grass biochar and inorganic fertilizer had an insignificant change on total yield and marketable and unmarketable fresh bulb yield of onion but biochar of grass and chat waste has shown a significant difference in agronomic characteristic and yield of the crop. The crop height was similar on the treatment of biochar grass and inorganic fertilizer, but it is lower in chat and controlled treatment. The biochar of grass and inorganic fertilizer has a similar effect on the marketable bulb yield of onion. Therefore, the application of biochar improves soil fertility status similarly with inorganic fertilizer, along with onion productivity. Based on P, K, N, CEC, EC, and SOM analysis, the supply of grass biochar to soils increased onion yield as equal as the supply of inorganic fertilizer but these parameters were lower in the chat treatment. Therefore, grass biochar can be used as a substitute for inorganic fertilizers which is easily available and prepared by the local farmers. During the preparation of biochar, in addition to using it as fertilizer, alternative energy sources (ethanol or syngas) production must be taken into consideration in the future. The nutritional content of onion based on biochar and inorganic fertilizer treatments needs to be investigated further in future research.

## Figures and Tables

**Table 1:** The composition and characterization of the biochar.

Parameters	Grass	Chat
pH	6.69 ± 0.4	5.1 ± 0.2
Electric conductivity (dS m^−1^)	0.38 ± 0.03	0.25 ± 0.02
Cation exchange capacity (cmolc kg^−1^)	29.9 ± 2.4	19.8 ± 1.3
Organic matter (OM)	6.7 ± 1.2	1.5 ± 0.07
Sodium	0.59 ± 0.03	0.27 ± 0.01
Potassium	9.90 ± 1.2	4.1 ± 1.1
Magnesium	8.1 ± 0.9	3.45 ± 0.9
Nitrogen	0.45 ± 0.02	0.24 ± 0.03
Sulfur	52.45 ± 3.45	10.4 ± 1.5
Phosphorus	6.34 ± 0.8	0.42 ± 0.06

**Table 2:** Soil pH, EC, and CEC analysis in the biochar and inorganic fertilizer treatment.

Treatment	pH^1^	EC^1^	CEC^1^
Wfb^b^	4.92 ± 0.8	0.2 ± 0.03	16.28 ± 1.9
Bc^b^	5.38 ± 0.9	0.23 ± 0.01	18.23 ± 2.1
Bg^a^	6.43 ± 1.2	0.42 ± 0.02	28.08 ± 3.8
If ^a^	6.74 ± 1.0	0.33 ± 0.03	25.36 ± 3.5

Bg = biochar grass; Bc = biochar chat; If = inorganic fertilizer; Wfb = control plot (free from biochar and fertilizer). ^1^Mean of the four-treatment pH, electric conductivity (EC), and cation exchange capacity (CEC). Treatments with the same letters were not significantly different at *p* ≤ 0.05.

**Table 3:** Soil property variation of elements within the treatments.

Treatment	% SOM	N	S	P
Wfb^b^	0.98 ± 0.04	0.11 ± 0.001	8.98 ± 1.4	0.66 ± 0.07
Bc^b^	1.03 ± 0.2	0.21 ± 0.01	9.94 ± 1.54	0.37 ± 0.05
Bg^a^	5.74 ± 0.45	0.38 ± 0.04	49.46 ± 6.7	5.66 ± 1.3
If ^a^	3.75 ± 0.21	0.35 ± 0.0.02	49.98 ± 6.9	5.67 ± 1.01

Bg = biochar grass; Bc = biochar chat; If = inorganic fertilizer; Wfb = control plot (free from biochar and fertilizer); SOM = soil organic matter; N = nitrogen; S = sulfur; P = phosphorus. Treatments with the same letters were not significantly different at *p* ≤ 0.05.

**Table 4:** Metallic ion effect on soil.

Treatment	Na	K	Mg
Wfb^b^	0.06 ± 0.001	2.86 ± 0.9	2.91 ± 0.2
Bc^b^	0.24 ± 0.01	3.68 ± 0.7	3.01 ± 0.9
Bg^a^	0.55 ± 0.02	9.27 ± 0.9	7.96 ± 0.6
If ^a^	0.64 ± 0.03	11.41 ± 1.1	7.17 ± 0.5

Bg = biochar grass; Bc = biochar chat; If = inorganic fertilizer; Wfb =  controlled plot (free from biochar and fertilizer); Na = sodium; K = potassium; Mg = magnesium. Treatments with the same letters were not significantly different at *p* ≤ 0.05.

**Table 5:** Treatment effects on plant maturity and plant height at different growth stages.

Treatment	70% maturity	PHI	PHII	PHIII	PHIV
	Mean ± SE	Mean ± SE	Mean ± SE	Mean ± SE	Mean ± SE
Wfb^b^	103.3 ± 0.31	35.1 ± 0.05	40.1 ± 0.05	45.0 ± 0.63	50.5 ± 0.03
Bc^b^	102.5 ± 0.33	36.0 ± 0.23	40.3 ± 0.01	46.6 ± 0.08	49.5 ± 0.14
Bg^a^	116.3 ± 0.33	37.1 ± 0.01	46.1 ± 0.01	52.7 ± 0.03	58.6 ± 0.08
If ^a^	117.3 ± 0.13	37.5 ± 0.02	45.0 ± 0.08	53.1 ± 0.02	60.0 ± 0.21

Bg = biochar grass; Bc = biochar chat; If = inorganic fertilizer; Wfb =  control plot (free from biochar and fertilizer); PHI = plant height at the initial stage; PHII = plant height at mid-stage; PHIII = plant height at the development stage; PHIV = plant height at a late stage. Treatments with the same letters were not significantly different at *p* ≤ 0.05.

**Table 6:** Effect of applied treatment on yield components.

Treatment	MY ton/ha	UNMY ton/ha	TY ton/ha
	Mean ± SE	Mean ± SE	Mean ± SE
Wfb^b^	17.51 ± 0.07	7.02 ± 0.02	24.53 ± 0.09
Bc^b^	16.30 ± 0.05	8.14 ± 0.02	24.44 ± 0.07
Bg^a^	28.24 ± 0.01	2.93 ± 0.02	31.17 ± 0.03
If ^a^	28.96 ± 0.02	2.82 ± 0.07	31.78 ± 0.09

Bg = biochar grass; Bc = biochar chat; If = inorganic fertilizer; Wfb =  control plot (free from biochar and fertilizer); MY = marketable yield; UNMY = unmarketable yield; TY = total yield. Treatments with the same letters were not significantly different at *p* ≤ 0.05.

## Data Availability

The data used to support the findings of this study are available from the corresponding author upon request.
